# Stalking and Intrusive Behaviors in Ghana: Perceptions and Victimization Experiences

**DOI:** 10.3390/ijerph17072298

**Published:** 2020-03-29

**Authors:** Heng Choon (Oliver) Chan, Lorraine Sheridan, Samuel Adjorlolo

**Affiliations:** 1 Teaching Laboratory for Forensics and Criminology, Department of Social and Behavioral Sciences, City University of Hong Kong, Hong Kong, SAR, China; 2 School of Psychology, Curtin University, Western Australia 6845, Australia; Lorraine.Sheridan@curtin.edu.au; 3 Department of Mental Health, University of Ghana, Legon P.O. Box LG 25, Accra, Ghana; sadjorlolo@ug.edu.gh; 4 Research and Grant Institute of Ghana, Accra, Ghana

**Keywords:** stalking, intrusive behavior, perception, victimization, Ghana

## Abstract

Most studies of stalking and other forms of intrusive behavior are conducted in the West. Little is known about the phenomenon in the African context. The present work represents the first dedicated stalking study conducted in Ghana. Based on a sample of 371 male and female university students, this study explored the gender distribution of overall perceptions and experiences, and frequency and duration of personal worst experiences of stalking and intrusive behavior. Several significant gender differences were noted. Females were generally more likely than males to perceive a range of intrusive activities as unacceptable. Females and males were equally likely to have experienced aggression and surveillance, and unwanted attention types of behaviors, while males were more likely than females to have experienced persistent courtship and impositions, and courtship and information seeking types of behaviors. In respect of their worst experience of intrusive behavior, females were more likely to report unwanted communications, aggressive courtship, property damage, and harassment of third parties, whilst males were more likely to have been threatened with harm. More than half of our participants (55.5%) were judged to have been stalked. Given the devastating nature and impact of stalking victimization, the findings may provide impetus to increase awareness of stalking in Ghana and add urgency to calls for anti-stalking legislation.

## 1. Introduction

Intrusive behavior that includes stalking has long been a serious societal problem. In recent years, it has attracted significant attention from academics, law enforcement agents, healthcare professionals, field practitioners, policy makers, and the general public. Most work on stalking and intrusive behavior has been conducted in Australia, the United Kingdom, and the United States. However, recent studies that sampled different and mostly understudied populations indicate that stalking is not uncommon elsewhere and may be universal [1,2,3,4,5,6]. 

Stalking is difficult to define but is generally accepted to represent a pattern of repeated, unwanted intrusion by one individual into the life of another, in a manner that causes distress, disruption or fear [7,8]. It should be noted that some behaviors that could be regarded as constituent of stalking may be similar to behaviors deemed acceptable within a courtship context. For instance, making telephone calls, sending gifts, or waiting outside an individual’s workplace are behaviors that may not seem too fearful or threatening in isolation within the context of courtship. However, if these behaviors are performed repeatedly against another individual who views them as unwanted, they can be threatening [9,10,11]. Stalking has been defined in various ways, including strict legal definitions that require the stalker to demonstrate intent and the victim to feel fear, or by broader definitions that include lists of constituent behavior [12,13,14]. 

Irrespective of the variation in defining stalking and its constituent activities, the negative effect on those victimized by such behaviors is clearly substantial. Victims of stalking often experience a wide array of psychological, physical, social, occupational, and financial costs resulting from their stalking experience [10]. For instance, victims may invest in additional security measures and socialize less in response to being stalked [15]. Victims and survivors have been found to have a poorer sociodemographic and psychosocial status after prolonged stalking victimization than controls [16].

In addition to studies of the actual experience of stalking, one research area that has received attention in recent years is perceptions of stalking. Exploring perceptions of intrusive behavior and stalking is important as studies have identified misconceptions that the general public holds about stalking behavior and appropriate responses to it. If left unaddressed, misconceptions may lead to a lack of demand for policy and social change [17]. This is particularly essential in jurisdictions that are yet to introduce anti-stalking related legislation. In this study, a sample of male and female Ghanaians was recruited to explore their perceptions and experiences of stalking and intrusive behaviors, in order to obtain insights from this rarely studied population. Stalking and intrusive behaviors have yet to be outlawed in Ghana.

### 1.1. Gender and Stalking

Personal intrusion, and stalking behavior in particular, is commonly viewed as a gendered offense. Research indicates that males are more likely to be the perpetrators, while females are more likely to fall prey to victimization [18]. Indeed, empirical studies overwhelmingly demonstrate that opposite-gender stalking is the most prevalent type of stalking [2,4,14,19,20,21]. A meta-analysis by Spitzberg [22] found that over 70% of stalkers were male and over 80% of victims were female. Nonetheless, same-gender stalking and female-male stalking are not unusual [18,23,24,25]. Although males may less be likely to self-identify as stalking victims and to report their victimization to the police [26], these factors do not explain the overall gendered pattern of stalking. 

Nicastro and colleagues [27] noted that the general observation concerning gender differences is more obvious when all subtypes of stalking are examined, and particularly when ex-intimate stalking is considered in isolation. The nature of stalking may be more violent when the stalker is an ex-intimate partner [28]. Differences have been found in the types of stalking behaviors engaged in by male and female stalkers. For example, Wood and Stichman [29] found that their sample of female university students were more likely than their male counterparts to report experience of the following stalking behaviors: being spied upon; receiving unsolicited phone calls and text messages; seeing the stalker lurking outside their home, school, or workplace; and seeing the stalker in places they should not be. Males were more likely to experience having the stalker vandalize their property or destroying something they loved. A recent study by Chan and Sheridan [2] of a large sample of Hong Kong young adults found that males were more likely than females to report stalking victimization to authorities, to experience the stalker contacting friends or family members to learn of their whereabouts, and to receive unsolicited phone calls from the stalker. For both men and women, studies tend to find that surveillance-oriented activities are the most frequently experienced stalking behaviors [30].

Empirical research that focuses on perceptions of stalking has largely aimed to identify behavior that the general public would regard as stalking behavior [1,31,32]. In a recent study of a large sample of Hong Kong young adults, significantly more females than males judged a range of intrusive activities as constituent of stalking [1]. McKeon and colleagues [33] reported that males more strongly endorsed problematic stalking myths than did females in their sample of Australian community members and police officers. Using a US college sample, Yanowitz [32] reported that females selected a larger number of activities as stalking behavior than did males, regardless of their personal stalking experiences. Also using a US college sample, Lambert and colleagues [17] noted that females were more inclined to judge that stalking occurred more regularly and was harmful to the victim than were males, again irrespective of their personal stalking victimization experiences. Nonetheless, there are studies that have failed to report any gender differences in perceptions of stalking behavior [34,35,36].

### 1.2. Stalking in Africa

Research on stalking in the African region has been scarce and little is known about the stalking phenomenon in the African context. To date, only a handful of empirical studies that sampled African populations have been published [6,37,38,39]. Pengpid and Peltzer [37] found that 58.2% of their sample of 268 South African female recipients of a protection order had experienced stalking victimization perpetrated by their intimate partner. Moreover, psychological abuse, physical violence, and stalking were significantly associated with intimate partner sexual assault. Analyzing the same dataset, Petzer and Pengpid [38] reported that the most frequent types of stalking behaviors experienced were the making of unwanted phone calls, spying, and attempting to communicate with the victim in other ways, against their wishes. Approximately one-third (34.3%) of the participants reported that they had received threatening messages through telephone calls.

In addition to the South African sample, Sheridan and colleagues [6,39] examined the perceptions and experiences of intrusive behaviors of 1734 young females from 12 countries, with 100 Egyptian females included. Sheridan and colleagues [39] reported that the intrusive activities perceived to be unacceptable by all Egyptian participants included “forced sexual contact”, “physically hurting someone you care about”, and “threatening to physically hurt you”. In relation to their actual victimization experiences, Sheridan and colleagues [6] found that the most frequently reported intrusive behaviors experienced by Egyptian females were “multiple telephone calls which you don’t want to receive” (86%), “finding out information about you (phone numbers, marital status, address, hobbies) without asking you directly” (86%), and “doing unrequested favors for you” (85%).

### 1.3. The Present Study

Ghana was the first sub-Saharan country to gain independence from European colonization on 6 March 1957. Ghana, formerly known as the Gold Coast due to an abundance of gold, is a West African state sharing borders with Cote d’Ivoire to the west, Burkina Faso to the north, Togo to the east and the Atlantic Ocean and the Gulf of Guinea to the south. The official language in Ghana is English. Given its historical ties, legislation and judicial practices in Ghana are primarily influenced by the British system although the Ghanaian criminal justice system has undergone significant changes since its independence [40]. 

Stalking is an old behavior, but a new crime. The first anti-stalking law was enacted in the United States in 1990 [41]. It is still not regarded as a crime in a majority of countries, particularly in most non-Western countries [6]. Ghana has not yet legislated against stalking and, like many African countries, has no formalized criminal justice response to stalking. Against this backdrop, the importance of this study is twofold. It is the first work to directly explore perceptions and experiences of stalking and intrusive behaviors in a sample of Ghanaians. This study aims to explore the gender distribution of perceptions and experiences, and the frequency and duration of participants’ worst experiences of stalking and intrusive behaviors. Perhaps more importantly, the findings of this study are anticipated not only to advance our knowledge on the topic, but also to inform practice in relation to social services for victims of stalking, and the development or refinement of public and social policies to help curb the phenomenon of stalking perpetration.

## 2. Methods

### 2.1. Participants and Procedure

A total of 371 participants were recruited within a Ghanaian public university, 50.7% female (*N* = 188) and 49.3% male (*N* = 183). Mean age was 24.09 years (*SD* = 5.03). There was a significant age difference between males (*M* = 25.43, *SD* = 5.64) and females (*M* = 22.78, *SD* = 3.95; *t* = 5.19, *p* < 0.001). A majority of the participants reported that they were single (60.7%) at the time of data collection. There are nine national public universities in Ghana, and students enrolled at public universities may receive government subsidies. Ethical approval was obtained from the third author’s institution. A selected number of course instructors were contacted for permission to administer the questionnaire to students during one of their scheduled classes. Informed consent was obtained from participants who were informed that their responses to the anonymous paper–pen questionnaire would be confidential and used only for research purposes. Participation in the study was voluntary, with no incentive provided. On average, participants took 20 minutes to complete the questionnaire. The response and cooperation rate for the survey was approximately 90%, with slightly over 400 individuals reached.

### 2.2. Measures

This study adopted a modified version of the “Stalking: International perceptions and prevalence” questionnaire (SIPPQ) originally developed by Sheridan and colleagues [42]. This measure was used as it has been used by multiple studies worldwide, and its use in this study allows comparisons between these sets of results. The original (42 intrusive incidents) and modified (47 intrusive incidents) versions of the measures have been used in at least nine previous studies [5,6], with samples collected from 13 countries (Armenia, Australia, Egypt, England, Finland, India, Indonesia, Italy, Japan, Portugal, Scotland, Singapore, and Trinidad). These samples were a mix of community and university student samples and none were representative.

The SIPPQ consisted of four sections, with the first section collecting the participants’ demographic characteristics (i.e., age, gender, marital status, current place of residence, and length of residence in Ghana). The second section comprised a measure of participants’ perceptions of a range of intrusive behaviors, some (but not all) of which are generally regarded as constituent of stalking. Participants read through a list of 47 intrusive behaviors and indicated those that they considered to be unacceptable from the perspective of being the target of each behavior. Some of the 47 behavioral items can also be found in two widely used stalking measures [Unwanted Pursuit Behavior Inventory [UPBI] [43]; Obsessive Relational Intrusion scale [ORI-P] [44]. For example, “Following you,” “Asking you for a date repeatedly,” and “Intercepting mail/deliveries”. Some of the behaviors can be regarded as routine and innocuous acts. For example, “A stranger engaging you in a conversation in a public place: such as at a bus stop or in a cafe”. The Cronbach’s α of the measure was 0.89 (males = 0.90, females = 0.87) in this study.

The third section comprised a measure of individual lifetime experiences of stalking and intrusive behaviors. Participants read through the same list of 47 behavioral items, but this time selected those they had personally experienced. The inclusion of innocuous behaviors indicated that it was implausible that any participant had never experienced any of the activities listed. The final section in SIPPQ provided a measure of actual stalking experiences, as opposed to simply asking about a range of intrusive acts. A definition of stalking was not provided to prevent potential priming effects. Participants were asked about their worst stalking/intrusive experience which was performed by a particular individual. Participants read through a list of 15 behavioral items and indicated the frequency (“Zero,” “1–4,” or “5+”) of any they had been subjected to during their worst stalking/intrusive experience. Next, the participants reported the duration of their worst stalking/intrusive experience (the options were “Less than two weeks,” “Two weeks to six months,” and “More than six months”). 

The 15 behavioral items were derived from Spitzberg and Cupach’s [10] meta-analysis that aimed to map the behavioral content of stalking. Given that stalking has been described as a constellation of behaviors [21], this study acknowledged that participants may have been subjected to activities other than the 15 listed. For an individual’s worst stalking/intrusive experience to be regarded as stalking in this study, the operational criterion of “Experiencing any combination of the 15 behaviors on at least 10 occasions for a minimum of two weeks” was adopted. This criterion was informed by Purcell and colleagues’ [9] findings that the lowest cut-off for intrusions to be perceived as problematic was two weeks, and Pathé et al.’s [18] widely used threshold of 10 occasions. In this study 10 occasions was determined by: Ten or more instances of “1–4” occurrences and/or two or more instances of “5+” occurrences, and/or a combination of five or more “1–4” occurrences and one or more “5+” occurrences.

A cluster analysis of the 47 behavioral items was performed by Sheridan and colleagues [39] based on responses from 1734 young females from 12 countries. Four clusters of stalking and intrusive behaviors emerged: (1) Aggression and surveillance (19 items), (2) Unwanted attention (7 items), (3) Persistent courtship and impositions (9 items), and (4) Courtship and information seeking (10 items). Two items failed to load on any of the behavioral clusters.

### 2.3. Analytic Strategy

In this study, chi-square analyses were performed to examine gender differences. This analytic method was adopted in view of the categorical nature of the variables of interest. The significance level was set at 0.05. Measures of association (i.e., phi and Cramer’s *V* coefficients) were used to interpret the strength of the relationships, and most importantly, to identify meaningful patterns. The effect size of phi (between two binary variables) and Cramer’s *V* (between two variables with at least three levels on one variable) were interpreted in terms of degrees of freedom. Using Cohen’s standards for cross-tabular effect size interpretation, an effect size of 0.16 and below was regarded as weak, between 0.17 and 0.28 as moderate, and 0.29 and above was considered as strong [45].

## 3. Results

### 3.1. Gender Distribution of Perceptions of Stalking and Intrusive Behaviors

As shown in Table 1, 28 of the 47 items (in four different behavioral clusters) were found to have generated agreement among more than 50% of participants that they were not acceptable. Behaviors most commonly regarded as not acceptable were: “Making death threats” (90.6%), “Criminal damage/vandalism to your property” (89.5%), and “Verbally abusing you” (89.5%); while the behaviors least likely to be considered unacceptable were: “Asking you out ‘as just friends’” (8.4%), “Sending or giving you gifts” (12.7%), and “Seeing him/her at the same time each day” (17.3%). Gender differences in perceptions of stalking and intrusive behavior were observed for 16 items, and in all cases females were more likely than males to consider the items unacceptable. The strength of these relationships was weak to moderate, ranging from 0.11 to 0.25.

### 3.2. Gender Distribution of Victimization Experiences of Stalking and Intrusive Behaviors

The lifetime victimization experiences of stalking and intrusive behaviors are presented in Table 2. Among 47 items, “Harming you physically” (89.2%) was the most commonly reported behavior experienced by the participants, followed by “Criminal damage/vandalism to your property” (88.9%) and “Making death threats” (88.1%); while “A stranger engaging you in a conversation in a public place (e.g., at a bus stop or in a café)” (28.8%), “Asking your friends, family, school, or work colleagues about you” (46.4%), and “Finding out information about you (phone numbers, marital status, address, hobbies) without asking you directly” (47.2%) were the behaviors reported least often. There were 11 significant gender differences noted. Interestingly, male participants were found to report higher frequencies of victimization on eight items, while female participants only reported higher frequencies than their male counterparts on three items. The strength of association in these gender differences was weak to moderate, ranging from 0.03 to 0.26.

### 3.3. Gender Distribution of Frequency of Worst Experience of Stalking/Intrusive Behaviors

Table 3 presents data summarizing participants’ reported worst experience of stalking/intrusive behaviors. Of the 15 behaviors asked about, “Phone calls, text messages, gifts, or letters” was the most frequently experienced (44.3% of respondents experienced this 5+ times from the same person), followed by “Declaring love for you” (34.1% reported 5+ experiences) and “Emails and/or messages on social media (e.g., Facebook) and other web-based communications (27.3% reported 5+ experiences). Conversely, “Damage to your property, wrecking things you care about, or hurting your pet” (1.8% reported 5+ experiences) and “Actually hurting people you care about” (3% reported 5+ experiences) were reported least often by participants. Significant gender differences were observed for four items. Relative to males, females reported more incidents of “Phone calls, text messages, gifts, or letters” (50.6% versus 37.5% 5+ experiences), “Emails and/or messages on social media (e.g., Facebook) and other web-based communications” (32.7% versus 21.5% 5+ experiences), and “Declaring love for you” (42.5% versus 25.3% 5+ experiences). In contrast, male participants reported experiencing “Threatening to hurt you” (5.5% versus 1% 5+ experiences) more often. The strength of these gender differences was relatively moderate, ranging from 0.15 to 0.20.

### 3.4. Gender Distribution of Duration of Perceived Worst Experience of Stalking and Intrusive Behaviors

Table 4 reveals the findings on the duration of the participants’ perceived worst experience of stalking/intrusive behaviors. Behaviors that the participants most often experienced for longer periods were “Phone calls, text messages, gifts, or letters” (39.4% persisted for more than 6 months), “Declaring love for you” (34.1% persisted for more than 6 months), and “Emails and/or messages on social media (e.g., Facebook) and other web-based communications” (22.7% persisted for more than 6 months); while the least persistent behaviors were “Damage to your property, wrecking things you care about, or hurting your pet” (1.6% persisted for more than 6 months) and “Actually hurting people you care about” (3% persisted for more than 6 months). 

Significant gender differences were found for seven stalking and intrusive behaviors. Relative to males, females experienced significantly longer durations of “Phone calls, text messages, gifts, or letters” (46.3% versus 32.2% persisting for more than 6 months), “Emails and/or messages on social media (e.g., Facebook) and other web-based communications” (27.1% versus 17.9% persisting for more than 6 months), “Damage to your property, wrecking things you care about, or hurting your pet” (2.1% versus 1% persisting for more than 6 months), “Harassing other people to upset you or find out information about you” (10.6% versus 6.5% persisting for more than 6 months), “Forcing you to talk to him/her” (19.6% versus 14.8% persisting for more than 6 months), and “Declaring love for you” (42.5% versus 25.3% persisting for more than 6 months). Interestingly, male participants were found to report a longer duration of perceived worst experience of stalking and intrusive behavior than female participants on “Threatening to hurt you” (5.5% versus 1.6% persisted for more than 6 months). The relationships were moderate in strength, with the values of Cramer’s *V* between 0.17 and 0.24.

Finally, 55.5% of the overall sample was judged to have experienced an episode of stalking according to the study criteria (i.e., experiencing any combination of the 15 behaviors on at least 10 occasions for a minimum of two weeks). Significantly more women (60.6%) had been stalked than men (50.3%; χ^2^ = 4.34, *p* = 0.03, Cramer’s *V* = 0.10).

## 4. Discussion

Stalking victimization is a global concern, with adverse consequences to physical, cognitive, psychological, and emotional well-being. Using a sample of 371 participants recruited within a Ghanaian public university, the present study examined perceptions and experiences of stalking and intrusive behaviors in Ghanaian males and females. The first dedicated empirical study of stalking and intrusive activity in Ghana, this work specifically explored the gender differences of overall perceptions and experiences, and the frequency and duration of perceived worst experiences of stalking and intrusive behaviors.

Notably, a number of significant gender differences were reported in the perceptions and experiences of stalking between males and females. Relative to males, significantly more females perceived the listed activities in the clusters of aggression and surveillance, unwanted attention, persistent courtship and impositions, and courtship and information seeking as unacceptable. Perhaps this may be related to the fact that females are more commonly the recipients of stalking and other forms of intrusive activity than are males [2,4,46]. The defensive attribution theory asserts that if an individual is making a judgment in a situation where they share some attributes with a potential victim of wrongdoing, then they are more likely to produce empathy-based responses [47]. Specifically related to the gender gap, Whitehead and Blankenship [48] claimed that females are usually more liberal in their perceptions of social issues and more supportive of progressive social causes; and thus, are more likely to demonstrate a greater willingness to extend rights to minority groups and females in general. As evident by opinion polling, the gender gap phenomenon largely transcends culture and nationality [49]. Nonetheless, mixed findings were reported in earlier studies. Some studies found that females are more likely to identify stalking and intrusive behaviors than males [1,17,50], while others failed to find this association [34,35,36].

Pertaining to the significant gender differences in the experience of stalking victimization, females and males were somewhat equally likely to have experienced aggression and surveillance, and unwanted attention types of stalking and intrusive behaviors. Interestingly, persistent courtship and impositions, and courtship and information seeking types of stalking and intrusive behaviors were reported significantly more frequently in males than females. These findings somewhat mirrored those reported in Chan and Sheridan’s [2] sample of Hong Kong young adults whereby males were significantly more likely than females to experience having the perpetrator contact their friends/family to learn about their whereabouts and make unsolicited phone calls to them at least once in their lifetime (i.e., courtship and information seeking). However, Wood and Stichman [29] found the opposite to be true where their US based female participants were more likely to experience surveillance and approach types of stalking and intrusive behaviors than were male participants. More cross-cultural empirical work is required to further examine the gender relationship in stalking victimization, especially if cultural norms and values play a vital role in these differences.

This study also examined the participants’ frequency and duration of perceived worst experiences of stalking and intrusive behaviors. Relative to males, females were reported to have experienced significantly more (i.e., more than five times) instances of unwanted attention, persistent courtship and impositions (i.e., “phone calls, text messages, gifts, or letters;” “emails and/or messages on social media [e.g., Facebook] and other web-based communications;” “declaring love for you;” and “damage to your property”). Further, these stalking and intrusive activities, along with some others (e.g., “harassing other people to upset you;” and “forcing you to talk to him/her”) were perpetrated for a longer duration (i.e., more than six months) towards females than males. Males, on the other hand, reported experience of physical threats (i.e., aggression and surveillance) significantly more frequently than their female counterparts, and these threats persisted for longer. 

Regardless of victim gender, the literature has consistently found that the longer the stalking continues, the greater the potential for psychological, social, and physical damage to the victims. For instance, Kamphuis and colleagues [51] found evidence that stalking duration is associated with post-traumatic stress symptoms in some stalking victims. A number of studies also indicate that the best predictor of stalking duration is the stalker–victim prior relationship, with rejected ex-partners found to be the most persistent, and strangers to be the least persistent [9,52]. Ex-intimate partners, especially those with personality disorders, are more likely to be charged with further stalking offenses [53]. It is therefore in the interests of victims to intervene and prevent a persistent stalking episode from developing via appropriate coping strategies. As asserted in many previous studies [3,4,54], the proactive coping approach (i.e., actively seeking formal and informal social support to respond to and end the stalking victimization) is arguably the most effective victim coping strategy.

The overall rate of stalking in this work was high (55.4%). This may be due to the form of measurement employed, as there was no requirement for the victim to have reported fear or threat. Also, as noted by Purcell and colleagues [55], prevalence rates for studies that determine stalking via the presentation of behavioral items appear to produce higher prevalence rates than those that employ a single rating question. Alternatively, it could be that Ghana genuinely has relatively high rates of stalking. A more nuanced measurement should be employed in future studies to help ascertain this.

A number of study limitations must be acknowledged. First, this study was limited by the use of self-reported data with a lack of depth in participants’ responses regarding their perceptions and victimization experiences. For instance, the SIPPQ does not measure the number of times that the 47 behaviors were performed. Thus, it is not surprising that participants judged aggressive behaviors as unacceptable, along with clearly criminal acts and some particular intimacy seeking behaviors. Given that stalking is often defined by persistence and repetition of behavior, future research may consider incorporating a frequency- and severity-based determination of unacceptability. Further, biases such as memory recall and social desirability are possible, which may subsequently result in an under-reporting of victimization experiences. Future studies should consider exploring additional victim characteristics, in conjunction with other circumstantial factors and obtain more comprehensive information to better understand the stalking victimization dynamics and overall experience. The study sample was not representative of the entire Ghanaian population. The sampling population was limited to a small and non-random sample in a public university in Ghana. The demographic background of students from public universities may differ from those from private universities. Hence, this study might not be able to fully reflect gender differences in perceptions and experiences of stalking and intrusive behavior of the wider Ghanaian population. Future research may consider recruiting a larger randomized sample to better understand stalking perceptions and victimization experiences in Ghana. Still, given that many previous studies have been based on international samples of students, the present work can be compared with these studies at least.

## 5. Conclusions

To the best of the authors’ knowledge, the present study is the first empirical work on the perceptions and experiences of stalking and intrusive behaviors in Ghana. Despite its limitations, this study has nevertheless offered solid groundwork for future studies on stalking and intrusive behaviors in Ghana. Given stalking has yet to be legislated in Ghana, this study is pertinent and timely to empirically demonstrate the devastating nature and impact of stalking victimization. It is hoped that stalking specific legislation will be encouraged in countries where it does not presently exist. For victims of stalking, prompt and effective intervention is essential before the problem escalates in severity and violence [e.g., resulting in sexual assault and homicide [56,57]. More specifically, given the gender differences associated with different perceptions and experiences of stalking and intrusive behaviors, it is wise for social service providers and mental health professionals to develop new intervention protocols or to refine existing plans to be more gender specific. This is because international research is consistently demonstrating that females and males both perceive and experience stalking and intrusive behaviors in a different manner.

## Figures and Tables

**Table 1:** Gender differences on perceptions of stalking and intrusive behavior (*N* = 371).

	Perceived as a Stalking and Intrusive Behavior					
	Overall	Male	Female	Gender Differences		
Items	*N* (%)	*N* (%)	*N* (%)	χ^2^	Phi	*p*
Cluster 1: Aggression and surveillance (19 items)						
01. Making death threats.	336 (90.6)	166 (90.7)	170 (90.4)	0.01	–0.01	*n.s.*
02. Criminal damage/vandalism to your property.	331 (89.5)	160 (87.9)	171 (81.0)	0.91	0.05	*n.s.*
03. Verbally abusing you	321 (86.5)	153 (83.6)	168 (89.4)	2.63	0.08	*n.s.*
04. Threatening to kill or hurt herself/himself if you refused to go out on a date with her/him.	316 (85.2)	152 (83.1)	164 (87.2)	1.28	0.06	*n.s.*
05. Hurting you emotionally (verbal abuse, ruining your reputation).	316 (85.2)	152 (83.1)	164 (87.2)	1.28	0.06	*n.s.*
06. Harming you physically.	307 (82.7)	150 (82.0)	157 (83.5)	1.24	0.06	*n.s.*
07. Forced sexual contact.	301 (81.4)	144 (78.7)	157 (84.0)	2.46	0.08	*n.s.*
08. Threatening to physically hurt you.	297 (80.3)	137 (75.3)	160 (85.1)	5.64	0.12	0.018
09. Physically hurting someone you care about.	291 (78.4)	141 (77.0)	150 (79.8)	0.41	0.03	*n.s.*
10. Secretly taking your belongings.	281 (75.7)	130 (71.0)	151 (80.3)	4.35	0.11	0.037
11. Confining you against your will.	281 (75.7)	136 (74.3)	145 (77.1)	0.4	0.03	*n.s.*
12. Trespassing on your property.	273 (73.6)	128 (34.5)	145 (77.1)	2.46	0.08	*n.s.*
13. Trying to manipulate or force you into dating her/him.	267 (72.0)	118 (64.5)	149 (79.3)	10.03	0.16	0.002
14. Acting in an angry manner when seeing you out with other people (e.g., your friends or romantic partners).	265 (71.4)	125 (68.3)	140 (74.5)	1.73	0.07	*n.s.*
15. Spying on you.	250 (67.4)	117 (63.9)	133 (70.7)	1.96	0.07	*n.s.*
16. Multiple telephone calls which you don’t want to receive.	244 (65.8)	115 (62.8)	129 (68.6)	1.37	0.06	*n.s.*
17. Taking photographs of you without your knowledge.	242 (65.2)	106 (57.9)	136 (72.3)	8.5	0.15	0.004
18. Intercepting mail/deliveries.	218 (58.9)	108 (59.3)	110 (58.5)	0.03	–0.01	*n.s.*
19. Following you.	217 (58.5)	84 (45.9)	133 (70.7)	23.57	0.25	< 0.001
Cluster 2: Unwanted attention (7 items)						
20. Sending you unwanted letters, notes, e-mail, or other written communications.	220 (59.3)	103 (56.3)	117 (62.2)	1.36	0.06	*n.s.*
21. Leaving unwanted items for you to find.	215 (58.0)	109 (59.6)	106 (56.4)	0.39	–0.03	*n.s.*
22. Refusing to accept that a prior relationship is over.	196 (52.8)	84 (45.9)	112 (59.6)	8.24	0.15	0.016
23. Standing and waiting outside your home.	139 (37.5)	67 (36.6)	72 (38.3)	0.11	0.02	*n.s.*
24. Giving or sending you strange parcels.	120 (32.3)	47 (25.7)	73 (38.8)	7.32	0.14	0.007
25. Standing and waiting outside your school or workplace.	109 (29.4)	52 (28.4)	57 (30.3)	0.16	0.02	*n.s.*
26. Driving, riding, or walking purposefully past your residence, school or workplace.	89 (24.0)	40 (21.9)	49 (26.1)	1.88	0.07	*n.s.*
Cluster 3: Persistent courtship and impositions (9 items)						
27. Someone at a social event such as a party asks you if you would like to have sex with him/her.	312 (84.3)	141 (77.5)	171 (91.0)	12.72	0.19	< 0.001
28. Someone engages you in an inappropriate personal and intimate discussion.	264 (71.2)	122 (66.7)	142 (75.5)	3.55	0.1	*n.s.*
29. Agreeing with your every word, even if you were wrong.	225 (60.6)	116 (63.4)	109 (58.0)	1.14	–0.06	*n.s.*
30. “Outstaying his/her welcome” in your home.	199 (53.6)	89 (48.6)	110 (58.5)	3.64	0.1	*n.s.*
31. “Wolf-whistling” in the street.	189 (50.9)	76 (41.5)	113 (60.1)	12.81	0.19	< 0.001
32. Making arrangements without asking you first (e.g., booking a table at a restaurant)	125 (33.7)	63 (34.4)	62 (33.0)	0.09	–0.02	*n.s*.
33. Asking you for a date repeatedly.	118 (31.9)	47 (25.7)	71 (38.0)	6.43	0.13	0.011
34. A stranger offering to buy you a drink in a café, restaurant, or bar.	113 (30.5)	50 (27.3)	63 (33.5)	1.68	0.07	*n.s.*
35. Sending or giving you gifts.	47 (12.7)	16 (8.7)	31 (16.5)	5.03	0.12	0.025
Cluster 4: Courtship and information seeking (10 items)						
36. Talking about you to mutual friends after meeting you just once.	137 (37.0)	63 (34.6)	74 (39.4)	0.89	0.05	*n.s*.
37. Visiting places because she/he knows that you may be there.	119 (32.2)	49 (26.9)	70 (37.2)	4.51	0.11	0.03
38. Changing classes, offices, or joining a new group to be closer to you.	92 (24.9)	36 (19.7)	56 (29.9)	5.23	0.12	0.022
39. Doing unrequested favors for you.	89 (24.0)	42 (23.0)	47 (25.0)	0.21	0.02	*n.s*.
40. Trying to get to know your friends in order to get to know you better.	88 (23.7)	34 (18.6)	54 (28.7)	5.27	0.12	0.022
41. Asking your friends, family, school, or work colleagues about you.	87 (23.5)	35 (19.2)	52 (27.7)	3.65	0.1	*n.s.*
42. Telephoning you after one initial meeting.	75 (20.2)	29 (15.8)	46 (24.5)	4.27	0.11	0.039
43. A stranger engaging you in a conversation in a public place (e.g., at a bus stop or in a café).	68 (18.3)	35 (19.1)	33 (17.6)	0.15	–0.02	*n.s.*
44. Seeing him/her at the same time each day.	64 (17.3)	28 (15.3)	36 (19.3)	2.04	0.07	*n.s.*
45. Asking you out “as just friends”.	31 (8.4)	14 (7.7)	17 (9.0)	0.24	0.03	*n.s.*
Cluster 5: Others (2 items)						
46. Coming round to visit you, uninvited, on a regular basis.	219 (59.2)	98 (53.8)	121 (64.4)	5.83	0.13	*n.s.*
47. Finding out information about you (phone numbers, marital status, address, hobbies) without asking you directly.	157 (42.3)	65 (35.5)	92 (48.9)	8.06	0.15	0.018

*Note.* Sample size (*N*), chi-square value (χ^2^), Phi coefficient (Phi), nonsignificant (*n.s.*), significant level (*p*).

**Table 2:** Gender differences on experiences of stalking and intrusive behavior (*N* = 371).

	Perceived as a Stalking and Intrusive Behavior					
	Overall	Male	Female	Gender Differences		
Items	*N* (%)	*N* (%)	*N* (%)	χ^2^	Phi	*p*
Cluster 1: Aggression and surveillance (19 items)						
01. Harming you physically.	330 (89.2)	154 (84.2)	176 (94.1)	9.53	–0.16	0.002
02. Criminal damage/vandalism to your property.	330 (88.9)	157 (85.8)	173 (92.0)	4.21	0.11	*n.s.*
03. Making death threats.	327 (88.1)	163 (89.1)	164 (87.2)	0.3	0.03	*n.s.*
04. Threatening to physically hurt you.	326 (87.9)	156 (85.2)	170 (90.4)	2.33	–0.08	*n.s.*
05. Intercepting mail/deliveries.	322 (86.8)	154 (84.2)	168 (89.4)	2.2	–0.08	*n.s.*
06. Physically hurting someone you care about.	319 (86.0)	153 (83.6)	166 (88.3)	1.69	–0.07	*n.s.*
07. Trespassing on your property.	316 (85.4)	147 (80.3)	169 (90.4)	7.49	–0.14	0.006
08. Forced sexual contact.	303 (81.9)	148 (81.3)	155 (82.4)	0.08	–0.02	*n.s.*
09. Confining you against your will.	300 (81.5)	142 (78.9)	158 (84.0)	1.62	–0.07	*n.s.*
10. Threatening to kill or hurt herself/himself if you refused to go out on a date with her/him.	290 (78.2)	150 (82.0)	140 (74.5)	3.06	0.09	*n.s.*
11. Secretly taking your belongings.	290 (78.2)	134 (73.2)	156 (83.0)	5.8	0.13	*n.s.*
12. Following you.	247 (66.6)	134 (73.2)	113 (60.1)	7.17	0.14	0.007
13. Trying to manipulate or force you into dating her/him.	250 (67.4)	128 (69.9)	122 (64.9)	1.08	0.05	*n.s.*
14. Taking photographs of you without your knowledge.	248 (66.8)	120 (65.6)	128 (68.1)	0.26	–0.03	*n.s.*
15. Verbally abusing you.	243 (65.7)	114 (62.6)	129 (68.6)	1.47	–0.06	*n.s.*
16. Spying on you.	225 (60.8)	110 (60.4)	115 (61.2)	0.02	–0.01	*n.s.*
17. Hurting you emotionally (verbal abuse, ruining your reputation).	213 (57.4)	102 (55.7)	111 (59.0)	0.41	–0.03	*n.s.*
18. Acting in an angry manner when seeing you out with other people (e.g., your friends or romantic partners).	193 (52.0)	93 (50.8)	100 (53.2)	0.21	–0.02	*n.s.*
19. Multiple telephone calls which you don’t want to receive.	176 (47.4)	98 (53.6)	78 (41.5)	5.41	0.12	0.02
Cluster 2: Unwanted attention (7 items)						
20. Leaving unwanted items for you to find.	306 (82.5)	143 (78.1)	163 (86.7)	4.7	–0.11	0.03
21. Giving or sending you strange parcels.	292 (78.7)	141 (77.0)	151 (80.3)	1.48	0.06	*n.s.*
22. Standing and waiting outside your school or workplace.	289 (77.9)	144 (78.7)	145 (77.1)	0.13	0.02	*n.s.*
23. Driving, riding, or walking purposefully past your residence, school, or workplace.	280 (75.5)	141 (77.0)	139 (73.9)	0.49	0.04	*n.s.*
24. Standing and waiting outside your home.	254 (68.5)	124 (67.8)	130 (69.1)	0.08	–0.02	*n.s.*
25. Refusing to accept that a prior relationship is over.	243 (65.5)	116 (63.4)	127 (67.6)	0.71	–0.04	*n.s.*
26. Sending you unwanted letters, notes, e-mail, or other written communications.	204 (55.0)	110 (60.1)	94 (50.0)	3.83	0.1	0.049
Cluster 3: Persistent courtship and impositions (9 items)						
27. “Wolf-whistling” in the street.	319 (86.0)	161 (88.0)	158 (84.0)	1.19	0.06	*n.s.*
28. Someone at a social event such as a party asks you if you would like to have sex with him/her.	303 (81.7)	147 (80.3)	156 (83.0)	0.44	–0.03	*n.s.*
29. Making arrangements without asking you first (e.g., booking a table at a restaurant).	260 (70.1)	131 (71.6)	129 (68.6)	0.39	0.03	*n.s.*
30. “Outstaying his/her welcome” in your home.	251 (67.7)	123 (67.2)	128 (68.1)	0.03	–0.01	*n.s.*
31. A stranger offering to buy you a drink in a café, restaurant, or bar.	236 (63.6)	133 (72.7)	103 (54.8)	12.82	0.19	< 0.001
32. Sending or giving you gifts.	223 (60.1)	115 (62.8)	108 (57.4)	1.13	0.06	*n.s.*
33. Someone engages you in an inappropriate personal and intimate discussion.	223 (60.1)	121 (66.1)	102 (54.3)	5.44	0.12	*0.02*
34. Asking you for a date repeatedly.	220 (59.3)	132 (72.1)	88 (46.8)	24.64	0.26	< 0.001
35. Agreeing with your every word, even if you were wrong.	210 (56.6)	97 (53.0)	113 (60.1)	1.9	–0.07	*n.s.*
Cluster 4: Courtship and information seeking (10 items)						
36. Changing classes, offices, or joining a new group to be closer to you.	292 (78.7)	144 (78.7)	148 (78.7)	0	0	*n.s.*
37. Seeing him/her at the same time each day.	280 (75.5)	137 (74.9)	143 (76.1)	0.07	–0.01	*n.s.*
38. Visiting places because she/he knows that you may be there.	242 (65.2)	122 (66.7)	120 (63.8)	0.33	0.03	0.03
39. Doing unrequested favors for you.	203 (54.7)	107 (58.5)	96 (51.1)	2.05	0.07	*n.s.*
40. Trying to get to know your friends in order to get to know you better.	198 (53.4)	107 (58.5)	91 (48.4)	3.78	0.1	*n.s.*
41. Asking you out “as just friends”.	195 (52.6)	104 (56.8)	91 (48.4)	2.64	0.08	*n.s.*
42. Talking about you to mutual friends after meeting you just once.	194 (52.4)	101 (55.5)	93 (49.5)	1.35	0.06	*n.s.*
43. Telephoning you after one initial meeting.	176 (47.4)	95 (51.9)	81 (43.1)	2.9	0.09	*n.s.*
44. Asking your friends, family, school, or work colleagues about you.	172 (46.4)	93 (51.1)	79 (42.0)	3.06	0.09	*n.s.*
45. A stranger engaging you in a conversation in a public place (e.g., at a bus stop or in a café).	107 (28.8)	66 (36.1)	41 (21.8)	9.18	0.16	0.002
Cluster 5: Others (2 items)						
46. Coming round to visit you, uninvited, on a regular basis.	233 (62.8)	118 (64.5)	115 (61.2)	0.44	0.03	*n.s.*
47. Finding out information about you (phone numbers, marital status, address, hobbies) without asking you directly.	175 (47.2)	89 (48.6)	86 (45.7)	0.31	0.03	*n.s.*

*Note.* Sample size (*N*), chi-square value (χ^2^), Phi coefficient (Phi), nonsignificant (*n.s.*), significant level (*p*).

**Table 3:** Gender differences on frequency of perceived worst experience of stalking and intrusive behavior (*N* = 371).

	Frequency of Perceived Worst Experience of Stalking and Intrusive Behavior								
	Overall		Male		Female		Gender Differences		
	1–4 times	5+ times	1–4 times	5+ times	1–4 times	5+ times	Cramer’s		
Items	*N* (%)	*N* (%)	*N* (%)	*N* (%)	*N* (%)	*N* (%)	χ^2^	*V*	*p*
01. Phone calls, text messages, gifts, or letters.	102 (30.5)	148 (44.3)	51 (31.9)	60 (37.5)	51 (29.3)	88 (50.6)	7.06	0.15	0.029
02. Emails and/or messages on social media (e.g., Facebook) and other web-based communications.	70 (23.6)	81 (27.3)	31 (21.5)	31 (21.5)	39 (25.5)	50 (32.7)	8.1	0.17	0.044
03. Trespassing on your property.	42 (14.7)	14 (4.9)	25 (17.4)	7 (4.9)	17 (12.0)	5 (3.5)	4.28	0.12	*n.s.*
04. Damage to your property, wrecking things you care about, or hurting your pet.	29 (10.2)	5 (1.8)	19 (13.2)	2 (1.4)	10 (7.1)	3 (2.1)	4.86	0.13	*n.s.*
05. Surveillance (following you, watching you, recording you).	83 (22.4)	36 (9.8)	40 (22.0)	16 (8.8)	43 (22.9)	20 (10.6)	1.31	0.06	*n.s.*
06. Ruining your reputation (sharing private pictures of you or sharing information about you, spreading lies about you).	64 (17.3)	32 (8.7)	35 (19.1)	18 (9.8)	29 (15.4)	14 (7.4)	4.23	0.11	*n.s.*
07. Threatening to hurt you.	51 (13.8)	12 (3.2)	34 (18.7)	10 (5.5)	17 (9.0)	2 (1.0)	14.63	0.2	0.006
08. Threatening to hurt people you care about.	40 (10.8)	12 (3.3)	23 (12.6)	7 (3.8)	17 (9.1)	5 (2.7)	4.68	0.11	*n.s.*
09. Actually hurting people you care about.	38 (10.3)	11 (3.0)	26 (14.3)	7 (3.8)	12 (6.4)	4 (2.1)	8.4	0.15	*n.s.*
10. Physically and/or sexually attacking you.	45 (12.2)	19 (5.1)	27 (14.8)	8 (4.4)	18 (9.6)	11 (5.9)	4.23	0.11	*n.s.*
11. Threatening to or actually hurting him/herself.	70 (18.9)	15 (4.0)	32 (17.6)	8 (4.3)	38 (20.2)	7 (3.7)	0.81	0.05	*n.s.*
12. Harassing other people to upset you or find out information about you.	51 (13.8)	32 (8.6)	31 (17.0)	12 (6.5)	29 (10.6)	20 (10.6)	4.73	0.11	*n.s.*
13. Forcing you to talk to him/her.	100 (27.0)	66 (17.9)	48 (26.4)	27 (14.8)	52 (27.7)	39 (20.7)	6.45	0.13	*n.s.*
14. Declaring love for you.	73 (19.7)	126 (34.1)	43 (23.6)	46 (25.3)	30 (16.0)	80 (42.5)	14.17	0.2	0.015
15. Getting other people to engage in any of the 14 behaviors listed above on their behalf.	71 (19.2)	28 (7.6)	39 (21.4)	12 (6.6)	32 (17.1)	16 (8.5)	2.29	0.08	*n.s.*

*Note.* Sample size (*N*), chi-square value (χ^2^), Cramer’s *V* coefficient (Cramer’s *V*), nonsignificant (*n.s.*), significant level (*p*).

**Table 4:** Gender differences on duration of perceived worst experience of stalking and intrusive behavior (*N* = 371).

	Duration of Perceived Worst Experience of Stalking and Intrusive Behavior										
	Overall			Male			Female			Gender	
	< 2 weeks	2 weeks	> 6 months	< 2 weeks	2 weeks	> 6 months	< 2 weeks	2 weeks	> 6 months	Differences	
		–6 months			–6 months			–6 months		χ^2^	
Items	*N* (%)	*N* (%)	*N* (%)	*N* (%)	*N* (%)	*N* (%)	*N* (%)	*N* (%)	*N* (%)	(Cramer’s *V*)	*p*
01. Phone calls, text messages, gifts, or letters.	65 (17.5)	101 (27.2)	146 (39.4)	45 (24.6)	50 (27.3)	59 (32.2)	20 (10.6)	51 (27.1)	87 (43.6)	14.95 (0.2)	0.002
02. Emails and/or messages on social media (e.g., Facebook) and other web-based communications.	114 (30.7)	69 (18.6)	84 (22.7)	75 (41.0)	30 (16.4)	33 (17.9)	39 (20.7)	39 (20.7)	51 (27.1)	21.24 (0.24)	0.001
03. Trespassing on your property.	196 (52.8)	42 (11.3)	17 (4.7)	104 (56.8)	25 (13.7)	9 (4.9)	92 (48.9)	17 (9.0)	8 (4.2)	10.04 (0.16)	n.s.
04. Damage to your property, wrecking things you care about, or hurting your pet.	214 (57.7)	30 (8.1)	6 (1.6)	116 (63.4)	19 (10.4)	2 (1.0)	98 (52.1)	11 (5.9)	4 (2.1)	13.33 (0.19)	0.01
05. Surveillance (following you, watching you, recording you).	144 (38.8)	85 (22.9)	37 (10.0)	83 (45.4)	41 (22.4)	16 (8.7)	61 (32.4)	44 (23.4)	21 (11.1)	8.28 (0.15)	*n.s.*
06. Ruining your reputation (sharing private pictures of you or sharing information about you, spreading lies about you).	178 (48.0)	64 (17.3)	33 (8.9)	93 (50.8)	35 (19.1)	18 (9.8)	85 (45.2)	29 (15.4)	15 (7.9)	8.00 (0.15)	*n.s.*
07. Threatening to hurt you.	195 (52.7)	51 (13.8)	13 (3.5)	95 (52.2)	34 (18.7)	10 (5.5)	100 (53.2)	17 (9.0)	3 (1.6)	15.27 (0.20)	0.004
08. Threatening to hurt people you care about.	201 (54.5)	40 (10.8)	13 (3.5)	104 (57.1)	23 (12.6)	7 (3.8)	97 (51.9)	17 (9.1)	6 (3.2)	6.3 (0.13)	*n.s.*
09. Actually hurting people you care about.	203 (55.0)	40 (10.8)	11 (3.0)	104 (57.1)	26 (14.3)	7 (3.8)	99 (52.9)	14 (7.5)	4 (2.1)	11.17 (0.17)	*n.s.*
10. Physically and/or sexually attacking you.	195 (52.7)	45 (12.2)	18 (4.9)	101 (55.5)	27 (14.8)	8 (4.4)	94 (50.0)	18 (9.6)	10 (5.3)	5.75 (0.13)	*n.s.*
11. Threatening to or actually hurting him/herself.	165 (44.6)	71 (19.2)	16 (4.3)	91 (50.0)	32 (17.6)	8 (4.3)	74 (39.4)	39 (20.7)	8 (4.2)	4.52 (0.11)	*n.s.*
12. Harassing other people to upset you or find out information about you.	172 (46.5)	53 (14.3)	32 (8.6)	95 (52.2)	31 (17.0)	12 (6.5)	77 (41.0)	22 (11.7)	20 (10.6)	10.98 (0.17)	0.027
13. Forcing you to talk to him/her.	114 (30.8)	99 (26.8)	64 (17.4)	70 (38.5)	48 (26.4)	27 (14.8)	44 (23.4)	51 (27.1)	37 (19.6)	12.84 (0.19)	0.015
14. Declaring love for you.	89 (24.1)	73 (19.7)	126 (34.1)	58 (31.9)	43 (23.6)	46 (25.3)	31 (16.5)	30 (16.0)	80 (42.5)	21.88 (0.24)	< 0.001
15. Getting other people to engage in any of the 14 behaviors listed above on their behalf.	166 (45.0)	72 (19.5)	27 (7.3)	88 (48.4)	39 (21.4)	12 (6.6)	78 (41.7)	33 (17.6)	15 (8.0)	5.31 (0.12)	*n.s.*

*Note.* Sample size (*N*), chi-square value (χ^2^), Cramer’s *V* coefficient (Cramer’s *V*), nonsignificant (*n.s.*), significant level (*p*), * *p* < 0.05, ** *p* < 0.01.
